# HIV status disclosure rate and reasons for non-disclosure among infected children and adolescents in Enugu, southeast Nigeria

**DOI:** 10.1080/17290376.2016.1226942

**Published:** 2016-09-01

**Authors:** A.C. Ubesie, K.K. Iloh, I.J. Emodi, N.S. Ibeziako, I.N. Obumneme-Anyim, O.N. Iloh, A.C. Ayuk, C.J. Anikene, J.E. Enemuo

**Affiliations:** ^a^ MBBS, MPH, FMCPaed, FWACP, is a Senior Lecturer at Department of Paediatrics, College of Medicine, University of Nigeria, Nsukka, Enugu, Nigeria; ^b^ Honorary Consultant at Department of Paediatrics, University of Nigeria Teaching Hospital, Ituku-Ozalla, Enugu, Nigeria; ^c^ MBBS, FMCPaed, FWACP is a Consultant Paediatrician at Department of Paediatrics, University of Nigeria Teaching Hospital, Ituku-Ozalla, Enugu, Nigeria; ^d^ MBBS, FMCPaed, FWACP, Professor of Paediatrics at Department of Paediatrics, College of Medicine, University of Nigeria, Nsukka, Enugu, Nigeria; ^e^ Honorary Consultant at Department of Paediatrics, University of Nigeria Teaching Hospital, Ituku-Ozalla, Enugu, Nigeria; ^f^ MBBS, FWACP, is an Associate Professor at Department of Paediatrics, College of Medicine, University of Nigeria, Nsukka, Enugu, Nigeria; ^g^ Honorary Consultant at Department of Paediatrics, University of Nigeria Teaching Hospital, Ituku-Ozalla, Enugu, Nigeria; ^h^ MBBS, FMCPaed, is a Consultant Paediatrician at Department of Paediatrics, University of Nigeria Teaching Hospital, Ituku-Ozalla, Enugu, Nigeria; ^i^ MBBS, FMCPaed, is a Senior Registrar at Department of Paediatrics, University of Nigeria Teaching Hospital, Ituku-Ozalla, Enugu, Nigeria; ^j^ MBBS, FMCPaed, is a Consultant Paediatrician at Department of Paediatrics, University of Nigeria Teaching Hospital, Ituku-Ozalla, Enugu, Nigeria; ^k^ MBBS, is a Senior Registrar at Department of Paediatrics, University of Nigeria Teaching Hospital, Ituku-Ozalla, Enugu, Nigeria; ^l^ MBBS, is a Senior Registrar at Department of Paediatrics, University of Nigeria Teaching Hospital, Ituku-Ozalla, Enugu, Nigeria

**Keywords:** *HIV*, *children*, *disclosure*, *caregivers*, *pediatric*, *rate*, *AIDS*, *Enugu*, *Southeast Nigeria*, *VIH*, *enfants*, *proactive*, *soignants*, *pédiatrie*, *taux*, *SIDA*, *Enugu*, *Sud-Est*, *Nigéria*

## Abstract

Aims: To determine the rate of HIV status disclosure, caregivers’ reasons for non-disclosure, and factors influencing disclosure among a sample of HIV-infected children in Enugu, southeast Nigeria. Methods: Data were collected prospectively via a questionnaire on HIV-infected children and their caregivers who visited the pediatric HIV clinic of the University of Nigeria Teaching Hospital between July 1, 2012, and June 30, 2013. The data analysis was performed using Statistical Package for the Social Sciences version 19 software. Results: Caregivers of 107 children (age 5–16 years; mean 10.1 ± 3.2 years) were enrolled in the study. There were 53 (49.5%) boys and 54 (50.5%) girls. HIV status had been disclosed to 31 (29%) of them. The major reason for non-disclosure was the child being considered too young. Age (*p *< .001), age at HIV diagnosis (*p *< .001) and baseline CD4 count (*p *= .008) were seen as significant predictors of HIV disclosure. Conclusions: There is a low rate of HIV disclosure to infected children, and it was found to be lower for younger children. We recommend improving efforts for disclosure counseling to caregivers in pediatric HIV clinics.

## Résumé

Objectifs: Déterminer le taux divulgation du statut VIH et les motifs de la non-divulgation fournis par le personnel soignant des enfants séropositifs à Enugu au Sud-Est du Nigéria. Méthodes: Les données ont été collectées de façon prospective auprès d'enfants infectés par le VIH et de leurs soignants au centre de prise en charge pédiatrique du VIH du Centre Hospitalier de l’Université du Nigéria entre le 1er juillet 2012 et le 30 juin 2013. L'analyse des données a été faite avec SPSS version19. Results: Les soignants de 107 enfants (âgés de 5 à 16 ans avec en moyenne 10.1 âgés de plus ou moins 3.2 ans) ont été inclus dans cette étude. Il y avait 53 (49.5%) hommes et 54 (50.5%) femmes. Le statut VIH a été divulgué à 31 (29%) des enfants. La principale raison de la non-divulgation était que l'enfant était “encore trop jeune”. L'âge (*p *< .001), l'âge au diagnostic du VIH (*p *< .001) et l’évaluation initiale du taux de CD4 (*p *= .008) étaient des prédicteurs significatifs de la divulgation du VIH. Conclusions: Il y a un faible taux de divulgation qui se révèle encore pire chez les jeunes enfants. Nous recommandons donc le renforcement du counseling sur la divulgation en faveur du personnel soignant des centres de prise en charge pédiatrique du VIH.

## Introduction

Most pediatric HIV infections occur perinatally; therefore the infected child is obviously unaware of his or her HIV status. ‘Disclosure’ implies revealing, making known, making public and/or sharing information on an issue (FMoH 2010). Disclosure of HIV serostatus occurs, thus, when the child is informed of his or her infection. The disclosure pattern ranges from non-disclosure, through partial disclosure to full disclosure (Namasopo-Oleja, Bagenda & Ekirapa-Kiracho 2015). Disclosure to HIV-infected children is a sensitive issue that must take the needs, feelings, and beliefs of the children and their parent(s)/caregiver(s) into account. Although one aspect of pediatric HIV disclosure refers to revealing HIV status to an infected child (Kallem, Renner, Ghebremichael & Paintsil 2011), rather than this being a one-time event, it is often a gradual process (Obermeyer, Baijal & Pegurri 2011). Other typical attributes of disclosure include ongoing discussion of health and health-related issues, starting the process early and using simple explanations of HIV disease for younger children, and in the case of older children educating about the virus’ nature and consequences (FMoH 2010).

The American Academy of Pediatrics advises that disclosure of HIV infection status to children and adolescents take into consideration age, psychosocial maturity, complexity of family dynamics, and clinical context (Turissini, Nyandiko, Ayaya, Marete, Mwangi, Chemboi, *et al*. 2013). Making HIV-infected children aware of their status is an issue of great concern for public health, partly because of the many proven benefits it brings for mental health, psychosocial development, caregiver well-being, treatment adherence, and future planning (Domek 2010). Poor adherence to HIV care and treatment has particularly poor implications for treatment failure and for increased morbidity and mortality. Caregivers of HIV-infected children, and the health-care professionals who treat them, are often reluctant to disclose status to infected children despite these potential benefits (COPA, 1999). Children who know their HIV status have been found to have higher self-esteem than infected children unaware of their status (COPA, 1999).

The African Network for the Care of Children Affected by HIV/AIDS (ANECCA) recommends that pediatric HIV disclosure start as early as at 5–7 years old (Tindyebwa, Kayita, Musoke, Eley, Nduati, Tumwesigye, *et al.* 2011). Similarly, the Nigeria National Guidelines for Paediatric HIV and AIDS Treatment and Care recommend starting the process in the same age range depending on the child’s level of comprehension and the consent of parents or caregivers (FMoH 2010). Despite these recommendations, studies have shown a widely varied rate of disclosure to HIV-infected children, ranging from 0% to 69% (Vreeman, Gramelspacher, Gisore, Scanlon & Nyandiko 2013). In Africa, reported rates of HIV status disclosure by caregivers to infected children ranged from 11% to 31.5% (Alemu, Berhanu & Emishaw 2013; John-Stewart, Wariua, Beima-Sofie, Richardson, Farquhar, Maleche-Obimbo, *et al.* 2013; Kallem *et al.* 2011; Livin, Bernadius, Rune, Gibson & Levina 2014; Turissini *et al.* 2013; Vreeman *et al.* 2013). Africa is home to over 90% of the world’s HIV-infected children younger than 15 years old (WHO 2014), and there are multiple reasons for the low rate of disclosure on the continent. A systematic review found the most commonly cited reason for non-disclosure among caregivers was that a child is not old enough or ready (Weiner 2007). The present study was therefore designed to determine the rate of HIV status disclosure, and reasons for non-disclosure among caregivers of HIV-infected children. The subject area was Enugu, southeast Nigeria.

## Methods

### Background/setting

This was a cross-sectional study of HIV-infected children aged 5–16 years, conducted at the pediatric HIV clinic of the University of Nigeria Teaching Hospital in Enugu, southeast Nigeria. This is a tertiary health facility and is the largest HIV treatment facility in the state of Enugu. The study population was 107 caregiver/child dyads.

### Consent

Informed consent was obtained from caregivers of the children and assent from children aged seven years and above.

### Data collection

A structured questionnaire was given to the caregivers of HIV-infected children, between July 1, 2012 and June 30, 2013. Questions asked included the child’s age and sex, age at HIV diagnosis, whether the child was aware of his/her HIV status and, if aware, at what age he/she was informed. Also sought were reasons for non-disclosure and the caregivers’ preferred age of disclosure. The baseline CD4, current CD4 (within the preceding 6 months) and viral load were obtained from the child’s medical records.

### Inclusion criteria

Caregivers and HIV-infected children visiting at the University of Nigeria Teaching Hospital pediatric HIV clinic were included in the study.

### Exclusion criteria

Caregivers who declined consent were excluded.

### Data analysis

The data analysis was conducted using Statistical Package for the Social Sciences software (SPSS version 19; IBM, Chicago, IL, USA). Descriptive statistics were used to report quantitative variables such as age, while qualitative variables such as socioeconomic class were reported using proportions. Chi-square and Fisher’s exact tests were used to test for a significant association of categorical variables. Fisher’s exact test was used if the expected number in a cell of a two-by-two table was less than five. The quantitative data were tested for normality using the Shapiro–Wilk normality test. Student’s *t*-test was used to compare means and 95% confidence interval (CI) reported. All reported *p*-values were two sided with *p *< .05 considered significant.

#### Ethical considerations

Ethical approval was obtained from the Health Research and Ethics Committee, University of Nigeria Teaching Hospital, Enugu.

## Results

### Characteristics of study participants

A total of 107 HIV-infected children were enrolled in the study. Mean age was 10.1 ± 3.1 years and there were 53 boys and 54 girls. Fifty-nine children (55.1%) were orphaned, and 45 (42.1%) were in World Health Organization (WHO) clinical stage 2 of the disease. Of all participants, 101 (94.4%) were on highly active antiretroviral therapy (HAART). Table 1 presents details of the participants’ characteristics.

**Table 1.** Characteristics of the study participants.

	*n* (%)
Age group (years)	
5–7	29 (27.1)
8–10	32 (29.9)
11–13	25 (23.4)
14–16	25 (19.6)
Sex	
Male	53 (49.5)
Female	54 (50.5)
Socioeconomic status	
Upper	12 (11.2)
Middle	52 (48.6)
Lower	43 (40.2)
Orphaned	
Yes	59 (55.1)
No	48 (44.9)
HAART	
Yes	101 (94.4)
No	6 (5.6)
WHO clinical stage	
1	28 (24.3)
2	45 (42.1)
3	31 (29.0)
4	5 (4.7)

### HIV status disclosure

HIV status had been disclosed to 31 of the 107 children (29%). Seventeen of 53 boys (32.1%) and 14 of 54 girls (25.9%) knew their status (*χ*^2^ **= 0.492, *p *= .528). The mean age at HIV disclosure was 11.52 ± 2.25 years. The mean ages of children who were aware or unaware of their status were 13.5 ± 18 years and 8.8 ± 2.5 years, respectively (*p *< .001). Status had been disclosed to 0%, 6.3%, 44% and 85.7% of the 29 children aged 5–7, 8–10, 11–13 and 14–16 years, respectively (*p *< .001), as given in Table 2. None of the 46 children aged **< 10 years knew their status, while 31 of 61 (50.8%) aged ≥10 years knew (Fisher’s exact test, *p *< .001). The mean age at HIV diagnosis was 6.39 ± 2.92 years among the disclosed group and 3.80 ± 2.74 years among the non-disclosed group (*p *< .001). Two of 12 children (16.7%) from the upper social class and 16 of 43 (37.2%) from the lower social class knew their status (odds ratio [OR]**= 2.96; 95% CI: 0.6–15.5; *p *= .19). Status had been disclosed to 20 of 59 (33.9%) orphans and 11 of 48 (22.9%) non-orphans (*χ*^2^ **= 1.55; *p *= .213). The mean age for caregivers’ suggested disclosure was 12.15 ± 2.20 years (11.71 ± 2.12 years and 12.33 ± 2.22 years among the disclosed and non-disclosed groups, respectively; *p *= .188).

**Table 2.** Factors affecting HIV-positive status disclosure to infected children.

Variables	HIV disclosure status		OR (95% CI)	*p*-Value
	Yes (%)	No (%)		
Age group (years)				
5–7	0 (0.0)	29 (100.0)	NA	<.001
8–10	2 (6.3)	30 (93.7)		
11–13	11 (44.0)	14 (56.0)		
14–16	18 (85.7)	3 (14.3)		
Orphaned				
Yes	20 (33.9)	39 (66.1)	0.6 (0.2 – 1.4)	.215
No	11 (22.9)	37 (77.1)		
Socioeconomic status				
Upper	2 (16.7)	10 (83.3)	3.0 (0.6 – 15.5)	.190
Lower	16 (37.2)	27 (62.8)		
Stages 1 and 2 disease				
Yes (%)	4 (12.9)	27 (87.9)	0.1 (0.1 – 0.4)	.088
No (%)	22 (28.9)	54 (71.1)		

Note: SD**= standard deviation, VL**= viral load, CI**= confidence interval, HAART**= highly active anti-retroviral therapy, NA**= not applicable.

### Factors preventing disclosure

Four of 33 (12.1%) children in WHO clinical stage 1 or 2 of the disease and 22 of 76 (28.9%) in stage 3 or 4 knew their status (Fisher’s exact test, *p *= .088), as given in Table 2. The mean baseline CD4 count among children who knew their HIV status was 501.93 ± 395.46/mm^3^ and 784.96 ± 601.43/mm^3^ among those who did not (*p *= .008). Table 3 gives details of the comparison of the mean current CD4 count, viral load and HAART duration. Twenty-four of 66 caregivers (36.4%) who had disclosed their child’s HIV status to another adult had also informed their children. Only seven of 41 (17.1%) had not disclosed the child’s status to someone else (*p *= .048). As shown in Table 4, among 73 caregivers (68.2%; 95% CI: 58.9–76.3), the most common reason for HIV non-disclosure was that the child was still too young.

**Table 3.** Mean variables of disclosed and non-disclosed groups of HIV-infected children.

Mean variables	HIV status disclosed		95% CI	*p*-Value
	Yes (mean ± SD)	No (mean ± SD)		
Age (years)	13.5 (1.8)	8.78 (2.5)	3.7 – 5.7	<.001
Age at diagnosis (years)	6.4 (2.8)	3.80 (2.7)	1.4 – 3.8	<.001
HAART duration (years)	5.2 (2.9)	4.6 (2.2)	0.4 – 1.8	.269
Baseline CD4 (cells/mm^3^)	501.93 (395.5)	784.96 (601.4)	35.0 – 531.1	.008
Current CD4 (cells/mm^3^)	701.74 (486.1)	866.72 (535.1)	84.6 – 414.6	.193
Current VL (copies/ml)	3500.06 (14177.5)	7416.96 (56800.6)	16649.1 – 24482.9	.706

**Table 4.** Reasons and barriers for non-disclosure of HIV status to infected children by their caregivers.

Reasons	*n* (%)	95% CI
Too young	73 (68.2)	58.9–76.3
Child may tell others	13 (12.1)	7.2–19.7
Afraid child may die	7 (6.5)	3.2–12.9
Suffer emotional disturbances	3 (2.8)	1.0–7.9

## Discussion

The present study showed that HIV status had been disclosed to only about one-third of the subject children. This figure is similar to that in studies conducted in Thailand (Oberdorfer, Puthanakit, Louthrenoo, Charnsil, Sirisanthana & Sirisanthana 2006), western Kenya (Vreeman, Scanlon, Mwangi, Turissini, Ayaya, Tenge, *et al.* 2014), and Ghana (Kallem *et al.* 2011), which reported disclosure rates of 21% to 30%. However, it is quite low compared with studies in higher income countries, where rates ranged from 57% to 100% (Bachanas, Kullgren, Schwartz, Lanier, McDaniel, Smith, *et al.* 2001; Blasini, Chantry, Cruz, Ortiz, Salabarría, Scalley, *et al.* 2004; Grubman, Gross, Lerner-Weiss, Hernandez, McSherry, Hoyt, *et al.* 1995). The lower rate of disclosure in the present study, and found in other African countries, might be due to fear of stigma and discrimination by family members and general communities. Fear of the child inadvertently disclosing the status to other people was the second most common reason for non-disclosure. Caregivers who have overcome stigma and disclosed the child’s HIV status to another adult were significantly more likely to disclose to their children. Although vigorous efforts have been put forth in combatting HIV/AIDS-related stigma and discrimination in sub-Saharan Africa, a great deal more work is needed (Apanga 2014). Strategies that address basic material needs, raise HIV/AIDS awareness, mobilize religious and community leaders, and involve the media are required to reduce such added struggles among HIV-infected children and their families (Grainger, Webb & Elliott 2001). While there are policies against HIV stigma and discrimination in Nigeria, about half the population still exhibits some forms of these behaviors (Dahlui, Azahar, Bulgiba, Zaki, Oche, Adekunjo, *et al*. 2015). This demonstrates why there is an urgent need to move from policy formulation to implementation to prevent stigma and discrimination from negatively affecting disclosure.

Another possible reason for the low disclosure rate found in the present study is poor implementation of WHO recommendations and guidelines for disclosure to HIV-positive children in Nigeria. Evidently there is insufficient counseling for parents/caregivers on the disclosure process. This was evident in both the late mean age at disclosure and the caregivers’ suggested mean disclosure age of 12.15 ± 2.20 years. Disclosure counseling for caregivers needs to be streamlined in comprehensive management of pediatric HIV/AIDS in Nigeria.

The mean age of HIV disclosure was 11.5 ± 2.3 years, compared with the 10.6 and 10.7 years reported in studies in western Kenya and northwest Ethiopia, respectively (Negese, Addis, Awoke, Birhanu, Muluye, Yifru, *et al.* 2012). As we expected, age was found to be a significant predictor of disclosure in the present study. While 85.7% of children aged 14–16 years were aware of their HIV status, none among those aged <10 years knew their status. In the Kenyan study, the disclosure rate also varied considerably by age (Vreeman *et al.* 2014). The Ethiopian study reported that age >10 years was significantly and independently associated with disclosure of HIV-positive status for infected children (Negese *et al.* 2012). The age for disclosure among HIV-infected children in this study and other African series thus fell short of the Nigerian (FMoH 2010), World Health Organization (2011) and ANECCA (Tindyebwa *et al.* 2011) recommendations. Also notable was that the most common reason cited by caregivers for non-disclosure was that the child was too young. We recommend that health-care providers commence discussions on child HIV disclosure with caregivers of perinatally infected children as soon as they are presented to pediatric HIV clinics. Other reasons caregivers mentioned for non-disclosure were consistent with those of studies in resource-limited countries, namely fear of emotional and health consequences, stigma and discrimination, and that the child would not keep the diagnosis confidential (Vreeman *et al.* 2013).

We found no significant difference in the proportion of boys versus girls whose HIV status has been disclosed to them. Although in most African cultures boys are traditionally expected to exhibit stronger coping mechanisms, this may not in fact be applicable. Disclosure was more likely to have occurred among children whose HIV was diagnosed at an older age. This may be because sick and older children are capable of asking questions about their illness following repeated hospital visits and hospitalizations. Severity may have played some role in disclosure, as our findings did show that the mean baseline CD4 count was significantly lower in children whose status had been disclosed to them than in those to whom it had not. Caregivers may have been compelled to disclose status to help the children’s coping mechanisms and support their adherence to treatment.

Contrary to findings in other studies (Ledlie 1999; Oberdorfer *et al*. 2006) where non-biologically related caregivers were more likely to disclose the child’s HIV-positive status than those biologically related, the present study showed no difference in the rate of disclosure among orphans and non-orphans. This could be due to the strong extended family systems in Nigeria that efficiently assume the role of caregiver when need arises.

Interestingly, the OR of HIV disclosure rate was threefold among children from lower socioeconomic class compared with their counterparts from the upper socioeconomic class. Obermeyer *et al.* (2011) argued that there are stronger incentives to disclose HIV status in settings wherein services are deficient and individuals rely on material support from their families and communities. Poverty, therefore, may be an incentive for disclosure by adults, and ultimately to the children as well. Our findings supported that caregivers who had disclosed a child’s HIV status to other adults were more likely to tell the child. Conversely, affluent families need minimal material support from others and can afford to maintain this level of secrecy for a longer time.

## Conclusions

The present study found a low rate of HIV status disclosure to HIV-infected children. Caregivers expressed concern that the children in their care were too young to know their status. Such tendencies underscore the need for inclusion of disclosure counseling of caregivers as part of comprehensive pediatric HIV management protocols in Nigeria.
